# Clinical efficacy and safety analysis of type A botulinum toxin in the treatment of adolescents with refractory overactive bladder

**DOI:** 10.1097/MD.0000000000038803

**Published:** 2024-07-05

**Authors:** Junhua Li, Wei Liu, Chenhao Tang, Huixian Pan, Chen Song

**Affiliations:** a Department of Urology, Hangzhou Third People’s Hospital, Hangzhou, P. R. China; b Department of Surgery, Zhejiang Medical & Health Group Hangzhou Hospital, Hangzhou, P. R. China

**Keywords:** adolescents, refractory overactive bladder, safety analysis, treatment efficacy, type A botulinum toxin

## Abstract

The objective of this study was to assess the clinical effectiveness and safety of type A botulinum toxin in the treatment of refractory overactive bladder in adolescents. We conducted a retrospective analysis of 37 adolescent patients with refractory overactive bladder who were treated at the Urology Department of Hangzhou Third People’s Hospital between January 2018 and August 2023. These patients received intravesical injections of type A botulinum toxin at a concentration of 10 U/mL, with an average of 20 injection points. We recorded changes in urination diaries and urodynamic parameters both before and 1 month after treatment. After 1 month of treatment, significant improvements were observed in several parameters, when compared to the pretreatment values. These included daytime frequency of urination (11.13 ± 6.45), average single void volume (173.24 ± 36.48) mL, nighttime frequency of urination (2.43 ± 0.31), urgency episodes (3.12 ± 0.27), initial bladder capacity (149.82 ± 41.34) mL, and maximum bladder capacity (340.25 ± 57.12) mL (all *P* < .001). After the first treatment, 5 patients had mild hematuria, 4 patients had urinary tract infection, and 1 patient had urinary retention, which was relieved after catheterization. No serious complications or adverse reactions were observed in other patients. The follow-up period ranged from 6 to 18 months, and the duration of efficacy varied from 2 to 8 months. Eight patients who initially had treatment failure achieved symptom relief after reinjection. In adolescents with refractory overactive bladder who do not respond well to conventional drug therapy, type A botulinum toxin can be administered safely and effectively. It significantly improves lower urinary tract symptoms and enhances the quality of life for these patients.

## 1. Introduction

Overactive bladder (OAB) is a prevalent urological condition among children and adolescents.^[1]^ In 2016, the International Children’s Continence Society characterized pediatric OAB as a clinical syndrome primarily marked by urgency, frequently accompanied by increased urination, nocturia, and enuresis, with or without urge incontinence, after ruling out urinary tract infections or evident pathological factors.^[2]^ Prolonged enuresis, frequent urination, and even urge incontinence not only result in psychological challenges such as diminished self-esteem and social anxiety in patients but also disrupt their education and daily life, causing considerable distress for both patients and their families. Mothers of OAB patients have reported elevated levels of psychological symptoms and traumatic experiences in their children.^[3]^ Presently, in clinical practice, OAB treatment often relies on the use of anticholinergic medications, such as oxybutynin, tolterodine, and solifenacin.^[3–5]^ Nevertheless, for certain patients, these conventional medications do not produce satisfactory outcomes. The American Urological Association defines patients with OAB as having refractory overactive bladder if, following an extended period of behavioral training, their OAB symptoms do not improve, or if they do not respond to anticholinergic drug treatment within 6 to 12 weeks (including inadequate symptom relief or intolerable adverse reactions).^[6]^

In the year 2000, the bladder wall injection of type A botulinum toxin as a treatment for neurogenic overactive bladder was initially reported. Subsequent to this treatment, improvements were noted in maximum detrusor pressure, bladder compliance, and bladder capacity.^[7]^ Currently, in Western countries, this approach has gained recognition as one of the available treatment options for refractory OAB, with a level of evidence of 1 and a grade A recommendation.^[8]^ In China, specific indications for this treatment have not yet been approved. Despite some small-scale studies demonstrating the effectiveness and safety of intravesical injection of type A botulinum toxin for OAB, there is currently no authoritative report regarding the treatment of refractory OAB in adolescents.

In this study, we performed a retrospective analysis of clinical data from 27 adolescent patients with refractory overactive bladder. Our aim was to assess the changes in urination diaries and bladder capacity before and after treatment with intravesical injections of type A botulinum toxin.

## 2. Materials and methods

### 2.1. Study population

We employed a retrospective analysis approach to select adolescents with refractory overactive bladder who sought care at the Department of Urology in Hangzhou Third People’s Hospital from January 2018 to August 2023 as the study population.

#### 2.1.1. Inclusion criteria

Age range of 12 to 18 years; diagnosed with refractory overactive bladder^[6]^; duration of the condition is ≥6 months; ineffectiveness or partial effectiveness of traditional medications like oxybutynin, tolterodine, or solifenacin, as well as intolerance to their side effects; signed informed consent for treatment.

#### 2.1.2. Exclusion criteria

Urination diaries suggesting an average single void volume >200 mL or urination frequency <8 times; bladder outlet obstruction, urethral stricture, and other organic diseases; presence of urinary tract stones, infections, tuberculosis, tumors; allergic to botulinum toxin; taking anticoagulant medications or having bleeding disorders; coexisting neurogenic bladder; coexisting lumbar-sacral spina bifida and other neurological conditions. According to the inclusion and exclusion criteria, a total of 37 patients were included in this study, including 17 boys and 20 girls, with an average age of 14.8 ± 5.1 years, as detailed in Table 1. All study subjects and their guardians provided informed consent for participation in this research. This study has been approved by the Medical Ethics Committee of our institution (Approval Number: Y-KL2021065).

**Table 1:** Basic information of 37 patients before and after treatment.

Number of patients	37
Average age (yr)	14.8 ± 5.1
Boys/girls	17/20
Duration (mo)	21.2 (6–48)
Degree of education (n, %)	
Senior middle school	16 (43.2%)
Junior middle school	17 (45.95%)
School dropout	4 (10.81%)
Complications (n, %)	
Hematuria	5 (13.51%)
Urinary tract infection	4 (10.81%)
Urinary retention	1 (2.7%)

### 2.2. Research methods

This study utilized a retrospective analysis approach. Before the treatment, patients and their parents were provided with an explanation of the potential side effects of the medication and the objectives of the trial. Upon obtaining informed consent from both the patients and their parents, intravesical injection treatment of type A botulinum toxin was administered.

### 2.3. Treatment implementation

The specific treatment procedures are as follows^[9]^: the patient is positioned in the lithotomy posture, standard disinfection is carried out, and local anesthesia is administered. Under the guidance of cystoscopy, a 6F long needle is used for intermittent injection of type A botulinum toxin (trade name: Hengli, Lanzhou Institute of Biological Products Co., Ltd., Gansu, China, specification: 100 U/bottle) into the detrusor muscle of the bladder. The dosage is maintained at 10 U/mL, with 100 U of the drug dissolved in 10 mL of normal saline. Injection sites are distributed at the bladder base, lateral walls, and dome, totaling 20 points, with each point receiving 0.5 mL of injection. The injection should be distributed as evenly as possible, with careful attention to avoiding the bilateral ureteral orifices, bladder trigone, and bladder neck. The injection depth should be approximately 2 mm beneath the mucosal layer to prevent bladder wall penetration. After the injection, the bladder is emptied, the cystoscope is withdrawn, and a urinary catheter is retained. Subsequent to the injection, ceftriaxone sodium 1 g is administered intravenously to prevent infection, once daily, for patients with no infection-related complications, for a duration of up to 48 hours. In the absence of specific circumstances, the urinary catheter is removed 3 days after the injection, and the treatment outcome is evaluated at 1 month posttreatment.

### 2.4. Evaluation of treatment efficacy

Both before and 1 month after treatment, we continuously recorded urinary diaries for a period of 3 days. We calculated various parameters, including daytime urination frequency, nighttime urination frequency, urgency episodes, and average single void volume. Additionally, we determined urodynamic parameters before and after treatment, which included initial bladder capacity and maximum bladder capacity.

### 2.5. Statistical analysis

The relevant data underwent statistical analysis utilizing SPSS 19.0 software. The normality of continuous variables was assessed using the Kolmogorov–Smirnov test. Normally distributed data were presented as mean ± standard deviation (mean ± SD), while nonnormally distributed data were presented as median and range Md (P_1_, P_2_). Group comparisons were carried out using the *t* test, with significance set at *P* < .05.

## 3. Results

All 37 patients successfully received the injection of botulinum toxin into the detrusor muscle. When compared to their pretreatment condition, significant improvements were observed in daytime urination frequency, average single void volume, nighttime urination frequency, urgency episodes, initial bladder capacity, and maximum bladder capacity at the 1-month posttreatment mark (all *P* < .001), as detailed in Table 2. Among these patients, 5 cases experienced mild Hematuria, which resolved within 2 to 3 days, and 4 patients developed Urinary tract infections, 1 patient had Urinary retention, which was relieved after catheterization; no other severe complications or adverse reactions were reported.

**Table 2:** Comparison of various parameters before and after treatment with type A botulinum toxin in 37 patients (mean ± SD).

Observational indicators	Pretreatment	Posttreatment	*P* value
Daytime frequency of urination	24.12 ± 8.73	11.13 ± 6.45	<.001
Average single void volume (mL)	132.66 ± 24.22	173.24 ± 36.48	<.001
Nighttime frequency of urination	6.32 ± 1.78	2.43 ± 0.31	<.001
Urgency episodes	7.12 ± 1.43	3.12 ± 0.27	<.001
Initial bladder capacity (mL)	108.89 ± 32.15	149.82 ± 41.34	<.001
Maximum bladder capacity (mL)	266.42 ± 56.58	340.25 ± 57.12	<.001

The follow-up period ranged from 6 to 18 months, with a median follow-up time of (11.4 ± 2.5) months. The treatment effect persisted for a duration spanning 2 to 8 months, with a median duration of 6.5 months. In response to the patients’ and their families’ preferences, 8 patients received repeat injections following treatment failure, resulting in symptom improvement comparable to the initial injection, with no reported adverse reactions.

## 4. Discussion

The traditional treatment approach for OAB typically starts with baseline voiding training and behavioral therapy. If these strategies prove ineffective, combination therapy involving medication becomes a second-line treatment option. In cases where patients remain unresponsive to these treatments, combination medication therapy may be considered.^[10]^ Among the available treatment options, behavioral therapy and anticholinergic drugs like tolterodine and oxybutynin are the primary clinical treatments for OAB. However, due to the notable side effects associated with anticholinergic drugs and challenges related to patient compliance, some OAB patients do not achieve the desired treatment outcomes. For these refractory OAB patients, intradetrusor injection of type A botulinum toxin into the detrusor muscle may provide unexpected therapeutic benefits.^[11]^

### 4.1. Maintenance of the efficacy of intravesical injection of type A botulinum toxin in the treatment of refractory OAB in adolescents

In this study, the efficacy of the initial injection of type A botulinum toxin in patients persisted for a period ranging from 2 to 8 months, with a median duration of 6.5 months. In cases where patients and their families were willing, 8 patients received repeat injections, and their symptoms continued to improve.

Research has indicated that intravesical injection of type A botulinum toxin for the treatment of refractory interstitial cystitis/bladder pain syndrome maintains its efficacy for approximately 6 to 12 months, and repeat injections demonstrate sustained effectiveness, with a more favorable long-term success rate compared to single injections.^[12]^ Moreover, there is evidence suggesting that repeated intravesical injections may lead to a cumulative effect.^[13]^ Thus, it is evident that multiple repeat injections of type A botulinum toxin can provide long-term efficacy. Nevertheless, the decision to opt for repeat injections should take into account factors such as the patient’s financial situation and their expectations regarding the benefits of repeated bladder injection therapy.

### 4.2. Safety of intravesical injection of type A botulinum toxin in the treatment of refractory OAB in adolescents

All patients in this study successfully completed the initial intravesical injection of type A botulinum toxin, with a total dose of 100 U. Despite a few patients experiencing mild gross hematuria and urinary tract infections, no overt symptoms of bladder perforation, voiding difficulties, urinary retention, or allergic reactions were observed. Furthermore, both hematuria and urinary tract infections improved within 2 to 3 days with the passage of time and appropriate treatment. Eight patients received repeat injections without any significant adverse reactions. It is evident that the intravesical injection of 100 U of type A botulinum toxin in adolescents is relatively safe, and repeat injections continue to provide benefits to patients.

### 4.3. Adverse reactions of intravesical injection of type A botulinum toxin in the treatment of refractory OAB in adolescents

The primary adverse reactions associated with intravesical injection of type A botulinum toxin typically encompass urinary tract infections (occurring in approximately 18% of cases), voiding difficulties (seen in around 9% of cases), and urinary retention (noted in approximately 3%–6% of cases), which tend to gradually resolve over time.^[11]^ In this study, there were 5 cases of hematuria (13.51%), 4 cases of urinary tract infection (10.81%), and 1 case of urinary retention (2.7%), which required indwelling catheterization and drug treatment. Other patients had no obvious dysuria and urinary retention symptoms, which were different from the relevant reported data. This discrepancy might be attributed to the operator’s skill level and the choice of injection sites. Additionally, the first follow-up in this study occurred at 1 month, and even if voiding difficulties had arisen, it’s possible that symptoms may have already improved by that time.

## 5. Conclusions

The findings from this study underscore that intravesical injection of type A botulinum toxin can offer significant short-term benefits for some adolescents dealing with refractory OAB characterized by unstable contractions of the bladder detrusor, especially when conventional drug treatments prove ineffective or only partially effective. Furthermore, repeated injections of type A botulinum toxin exhibit a high degree of safety and efficacy. However, it’s important to acknowledge the limitations of this study, including challenges related to patient compliance with repeated injections, a relatively small sample size, and a lack of continuous observation. Consequently, it remains difficult to comprehensively assess the effects of repeated injections on a larger scale, which constitutes one of the study’s constraints. Additionally, there is limited evidence regarding the optimal timing and duration of repeated injections, necessitating further research for validation. In future studies, we intend to expand the sample size and delve deeper into the exploration of the duration of efficacy among patients who undergo multiple repeated injections, as well as investigate the correlation between the interval of repeated injections and their effectiveness.

## Author contributions

**Data curation:** Junhua Li.

**Writing—original draft:** Junhua Li.

**Formal analysis:** Wei Liu, Chenhao Tang.

**Software:** Wei Liu.

**Conceptualization:** Huixian Pan, Chen Song.

**Investigation:** Huixian Pan.

**Funding acquisition:** Chen Song.

**Writing—review & editing:** Chen Song.
